# Cardiac manifestations in hyperthyroidism

**DOI:** 10.31083/j.rcm2304136

**Published:** 2022-04-11

**Authors:** Alberto Navarro-Navajas, José David Cruz, Nicolas Ariza-Ordoñez, Helman Giral, Jorge Palmezano, Adrián Bolívar-Mejía, Quindo Santana, Ricardo Fernandez, Luisa Durango, Clara Saldarriaga, Juan Camilo Mateus, Diego Garnica, José Guillermo Sarta-García, Fernando Lizcano, Carlos Andrés Tapias

**Affiliations:** ^1^Escuela de medicina, Universidad del Bosque, Fundación Cardioinfantil, 110131 Bogota, Colombia; ^2^Escuela de medicina y ciencias de la salud, Universidad del Rosario, Fundación Cardioinfantil, 110131 Bogota, Colombia; ^3^Escuela de medicina, Universidad Pontificia Bolivariana, Cardio-VID Clinic, 050010 Medellin, Colombia; ^4^Departamento de cardiología, Cardio-VID Clinic, 050010 Medellin, Colombia; ^5^Programa de falla cardiaca, Cardio-VID Clinic, 050010 Medellin, Colombia; ^6^Escuela de medicina y ciencias de la salud, Universidad del Rosario, Fundación Cardioinfantil, 110131 Bogota, Colombia; ^7^Centro de Investigación Biomédica Universidad de La Sabana (CIBUS), Fundación Cardioinfantil, 110131 Bogota, Colombia; ^8^Departamento de electrofisiología, Centro Internacional de Arritmias, Fundación Cardioinfantil, 110131 Bogota, Colombia

**Keywords:** hyperthyroidism, cardiovascular disease, heart failure

## Abstract

Thyroid hormones have a fundamental impact on cardiac function that is mediated by genomic and nongenomic effects, alterations that condition physiological repercussions that lead to changes in frequency, contractility, rhythm and cardiac output as well as an increase in the incidence and prevalence of different cardiovascular diseases. This document presents an updated review of the implications that hyperthyroidism has in different cardiac conditions, including its importance in the evaluation of perioperative cardiovascular risk.

## 1. Introduction

Thyroid hormones are essential in energy homeostasis, functioning as transcription factors. At the cardiovascular level, they have a substantial impact on the contractile apparatus and the sarcoplasmic reticulum of the myocardium through changes in the gene expression of several of its components, thus having effects on frequency, rhythm and cardiac output [1].

Hyperthyroidism is a pathological state in which the thyroid gland synthesizes and secretes an excess of thyroid hormone [2]. Given their systemic effects, thyroid hormones cause alterations in multiple organs, primarily in the cardiovascular system [1]. Hyperthyroidism is characterized by an increase in the synthesis and secretion of thyroid hormones by the thyroid gland, while thyrotoxicosis refers to the clinical syndrome derived from the excess of circulating thyroid hormones, regardless of the source (endogenous, both thyroid and nonthyroid, and exogenous) [3].

In this article, a systematic search of information was performed in the following databases: Index Medicus/MEDLINE (www.pubmed.com), Scopus (www.scopus.com), SciELO (www.scielo.org), IMBIOMED (www.imbiomed.com) and LILACS (www.bireme.br). The terms used were (in English Medical Subject Headings, MeSH; in Spanish *Descriptores en Ciencias de la Salud, DeCS*) «hyperthyroidism», «cardiovascular risk», «heart failure», «acute myocardial infarction», «atrial fibrillation», «thyroid storm»; «hipertiroidismo», «riesgo cardiovascular», «falla cardiaca», «infarto agudo de miocardio», «fibrilación auricular», «tormenta tiroidea». The search parameters included articles in English, Spanish and Portuguese available up to 2020. The main aspects of the cardiac manifestations of hyperthyroidism, its physiopathology and clinical approach are reviewed, with the aim of drawing attention to the relationship between hyperthyroidism and the evolution of heart diseases.

## 2. Thyroid physiology and its action on the cardiovascular system

Thyroid hormones influence the differentiation, growth and energy metabolism of almost all cells and tissues [4]. Consequently, these factors have an important impact on the energy homeostasis of the heart, and their excess leads to a hypermetabolic state [4, 5].

The thyroid gland produces 2 hormones: thyroxine (T4) and triiodothyronine (T3). T4 is the hormone most produced by and secreted from the thyroid gland (80–90%); however, with a T4:T3 ratio of 4:1, T3 has the greatest biological potency [5, 6]. The affinity of thyroid receptors for T3 is 10 times greater than that of thyroid receptors for T4 [1, 4].

The thyroid axis is formed and regulated by a negative feedback circuit that involves the hypothalamus, pituitary gland and thyroid gland. The hypothalamus secretes thyrotropin-releasing hormone (TRH), which stimulates the pituitary gland to release thyroid-stimulating hormone (TSH) [1, 5]. The latter activates the thyroid gland to produce and release thyroxine (T4) and triiodothyronine (T3) [4]. Increased thyroid hormone production normally inhibits the secretion of TRH and TSH in the hypothalamus and pituitary gland, respectively [4].

Another system involved in the functioning and regulation of the thyroid axis is deiodinases. These strictly control the intracellular concentrations of thyroid hormones by catalyzing the removal of iodine atoms in the phenolic ring (outer ring) or in the tyrosyl ring (inner ring) of T3 and T4 [6]. The conversion of T4 (mainly peripheral) to T3 is performed by the action of these enzymes, which are expressed in different tissues, depending on the age and different needs of each organ [6, 7]. There are 3 types of deiodinase. Type 2 (D2) converts T4 to T3 through deiodination of the outer ring. It is expressed in the central nervous system, skeletal muscle, brown adipose tissue and thyroid. Type 3 (D3) deactivates T4 and T3 by removing the iodine atom from the inner ring to produce reverse triiodothyronine (T3r) or diiodothyronine (T2) (respectively), which are inactive forms or do not have the genomic action of T3. It is found mainly in the brain, pancreas and placenta. Finally, deiodinase type 1 (D1), expressed in the liver, kidneys and thyroid, catalyzes the action of both types 2 and 3 and is thus capable of producing T3 and T3r [6, 7].

In cardiomyocytes, T3 enters through membrane transporters or is produced in the cell through the conversion of T4 to T3. The most important deiodinases are D2 and D3, which are of vital importance for the regulation of thyroid hormone levels and action in cardiac tissue [1, 5].

**Fig. 1.**
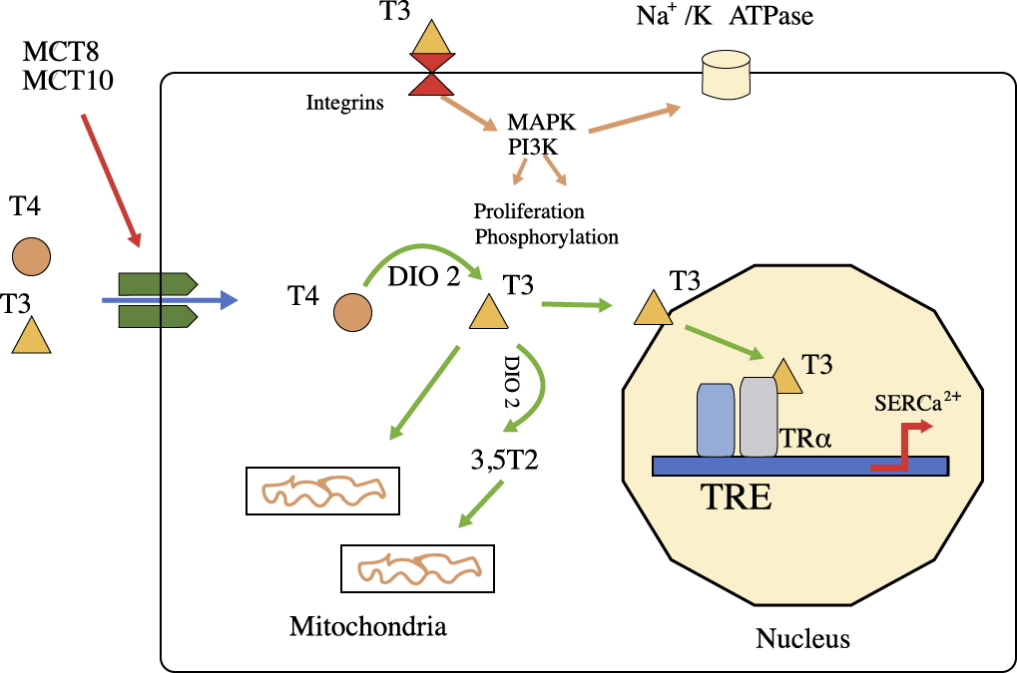
**The function of thyroid hormones**. T4 and T3 can enter cells (such as cardiomyocytes) by passive diffusion or through the action of transporters (MCT8 and MCT10). T4 is converted to T3 by deiodinase 2 (DIO2), and T3 enters the nucleus to modify the expression of specific genes. In the heart, thyroid hormones regulate the protein-encoding genes sarcoplasmic reticulum ${\mathrm{Ca}}^{2+}$ ATPase (SERCa2) and α-myosin heavy chain, among others. T3 can exert its function on the mitochondria by increasing the activity of proteins that increase energy in cells. Some metabolites, such as 3,5T2, can increase thermogenesis. T3 exerts nongenomic functions through interactions with membrane proteins such as integrins, modifies the function of mitogen-activated protein kinases (MAPKs) and regulates ion channels in the plasma membrane. Author FL and JCM.

The mechanisms of action of thyroid hormones can be classified into 2 categories: genomic and nongenomic [8]. The first is mainly mediated by T3, which binds to nuclear thyroid hormone receptors (belonging to the family of steroid receptors), which in turn bind to thyroid hormone response elements in the promoter regions of target genes [1, 9, 10]. With their binding to T3, nuclear receptors induce or repress the transcription of several genes at the cardiac level (see Table 1). Genes regulated by thyroid hormones involve structural and regulatory proteins of cardiac function. Long-term exposure to high levels of T3 can increase cardiac protein synthesis, leading to cardiac hypertrophy and dysfunction (Fig. 1) [8].

**Table 1.** **Genomic effects on cardiac muscle**.

Negatively regulated genes	Positively regulated genes
β-myosin heavy chain	α-myosin heavy chain
Phospholamban	${\mathrm{Ca}}^{2+}$ ATPase of the sarcoplasmic reticulum
Catalytic subunit of adenyl cyclase	${\mathrm{Na}}^{+}$/$K^{+}$ ATPase
${\mathrm{Na}}^{+}$/${\mathrm{Ca}}^{2+}$ exchanger	β1 adrenergic receptor
Thyroid hormone receptor α1	voltage-gated $K^{+}$ channels

Taken from Osuna P, Udovcic M, Sharma M. Hyperthyroidism and the Heart. Methodist DeBakey Cardiovascular Journal. 2017; 13: 60–63.

In this way, changes such as the reduction in phospholamban and increase in the ${\mathrm{Ca}}^{2+}$ ATPase pump of the sarcoplasmic reticulum improve myocardial relaxation. The increased expression of the faster contractile isoforms of the myosin heavy chain (α isoforms) contributes to improving systolic function [8].

Nongenomic actions cause rapid changes in several ion channels of the myocyte membrane (sodium, potassium, calcium), in the rate of actin polymerization and in several intracellular signaling pathways of cardiac and vascular smooth muscle cells [11, 12]. T3 also increases the rates of depolarization and repolarization of the sinoatrial node, thus increasing the heart rate [11, 12].

Consequently, thyroid hormones have positive inotropic and chronotropic effects on the heart. Both mechanisms (genomic and nongenomic) work together to maintain cardiac function and hemodynamic balance. However, elevated and prolonged exposure to thyroid hormones leads to cardiovascular imbalances [13].

### 2.1 Hemodynamic repercussions of hyperthyroidism

The cardiovascular system is sensitive to small variations in thyroid hormone concentrations; therefore, thyroid pathological states have predictable and clinically evident repercussions on the function of the heart and peripheral vasculature. In the hyperthyroid state, there are several mechanisms that lead to a hemodynamic state characterized by increased cardiac output (50% to 300% greater than that for healthy subjects) [1, 14].

### 2.2 Action on catecholamine sensitivity

In thyrotoxicosis, there is an increase in the density of β-adrenergic receptors, leading to greater sensitivity of the tissue to catecholamines. There is an increase in heart rate at rest, blood volume, systolic volume, myocardial contractility and diastolic function. In this way, it results in a hyperdynamic cardiovascular state characteristic of hyperthyroidism, which is why important therapeutic effects can be observed with the use of β-blockers in hyperthyroid patients [8, 15].

### 2.3 Effect on the renin-angiotensin-aldosterone (RAA) axis

**Fig. 2.**
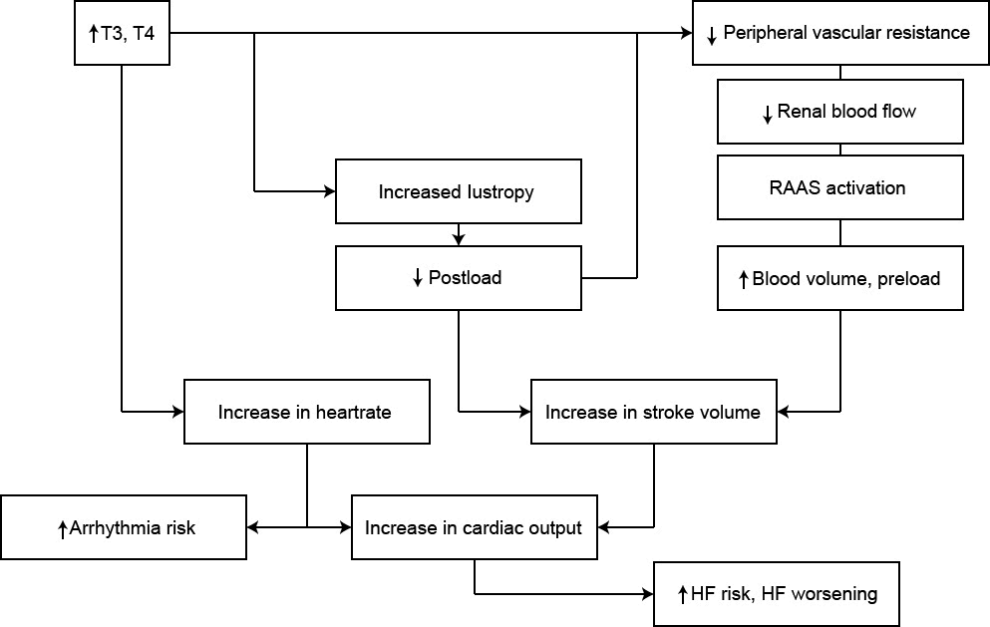
**Pathophysiological changes leading to a hyperdynamic circulatory state in hyperthyroidism**. Adapted from Vargas Uricoechea *et al*. [1].

In hyperthyroidism, there is a reduction in peripheral vascular resistance resulting in a decrease in renal perfusion pressure, leading to the activation of the RAA axis, which causes the increased renal reabsorption of sodium and water. In this way, there is an increase in blood volume, in preload and, finally, in systolic volume or cardiac output [15, 16] (Fig. 2, Ref. [1]). Additionally, T3 promotes hepatic angiotensin synthesis, renin synthesis at the cardiac level and a greater amount of angiotensin II receptors in the myocardium [1]. These hemodynamic changes cause stretching of the atrial fibers that trigger the secretion of atrial natriuretic peptide (ANP), which causes more vasodilation. These changes suggest a central role of the RAA axis in cardiac hypertrophy induced by thyroid hormones [8].

All the aforementioned alterations imply an increase in the risk of different cardiovascular diseases in the short, medium and long terms in the presence of an abnormal and disproportionate increase in thyroid effector hormones, which is why it is important to evaluate the impact of these changes at the cardiac level (Table 2).

**Table 2.** **Cardiac manifestations of hyperthyroidism**.

Cardiovascular disease	Considerations
Coronary artery disease	It can manifest as a chronic coronary syndrome, unstable angina or acute myocardial infarction with or without ST elevation.
	It increases the risk of atherosclerotic disease but also the risk of type 2 acute myocardial infarction and myocardial infarction without coronary obstructive lesions.
	Treatment depends on the pathophysiological mechanism found.
Heart failure	It increases the risk of developing heart failure with reduced or preserved ejection fraction.
	Left ventricular systolic dysfunction is potentially reversible with antithyroid treatment.
	Tachycardiomyopathy is usually a causal pathophysiological mechanism.
	After improvements in cardiac function, the continuity of the medications established for the management of heart failure should be reevaluated.
Arrhythmias	The most frequent manifestation is sinus tachycardia.
	There is an increased risk of atrial fibrillation, ventricular arrhythmias, syncope and sudden death.
	The presence of tachyarrhythmias can precipitate the development of tachycardiomyopathy.
	For atrial fibrillation, the indication for anticoagulation depends on the CHA2DS2-VASC score.
	Antiarrhythmic drugs such as amiodarone are thyrotoxic and should be avoided or used with caution in patients with thyroid disease.
Perioperative cardiovascular risk	Uncontrolled hyperthyroidism increases the risk of adverse cardiovascular outcomes in the perioperative period (coronary events, arrhythmias and decompensation of preexisting heart failure).
	Prior to elective surgery, the hyperthyroid state should always be controlled.
	Strict monitoring of the hemodynamic status should be performed in the intra- and postoperative periods.
Echocardiographic alterations	Subclinical stage: increase in LV parietal thickness, normal or slight increase in ejection fraction, decrease in longitudinal strain, alteration of diastolic function due to type I relaxation disorder, increase in isovolumic relaxation time, and increase in deceleration time.
	Clinical stage: LV hypertrophy or dilation, mitral valve prolapse, mitral insufficiency, tricuspid insufficiency, increased or decreased ejection fraction, increased LV filling pressures, dilation of the left atrium, and increased systolic pressure in the pulmonary artery.

Source: the author.

## 3. Hyperthyroidism and coronary artery disease

### 3.1 Hyperthyroidism as a risk factor for coronary artery disease

The high burden of morbidity and mortality caused by coronary artery disease has led to great efforts to identify risk factors susceptible to modification, which allow changing the natural history of the disease and its outcomes [17]. Alterations in thyroid function are one of these [18, 19]. In the HUNT study (the Trøndelag Health study), in which more than 65,000 adults participated, with an average follow-up of 12 years, both subclinical hyperthyroidism (defined by low or suppressed levels of TSH in the presence of normal free thyroxine and triiodothyronine levels) and frank hyperthyroidism were related to higher mortality from coronary events, particularly in the female population [20]. Likewise, in the Rotterdam cohort, with almost 10,000 people followed for an average of 8.8 years, the multivariate analysis showed that elevated levels of free T4 were associated with a higher coronary calcification index, higher rate of cardiovascular atherosclerotic events and higher mortality from cardiovascular events [21]. In a similar study with a decade-long follow-up of more than 80,000 Danish patients with hyperthyroidism, a 2- to 4-fold higher mortality from any cause and a 2- to 3-fold higher acute myocardial infarction rate were observed for those with hyperthyroidism [22]. Recently, Beyer *et al*. [23] performed a retrospective analysis of 745 patients who underwent coronary angiography, dividing them into 3 categories based on their thyroid profile: frank hyperthyroidism, subclinical hyperthyroidism and euthyroidism. The researchers noted that patients with hyperthyroidism had a higher degree of coronary stenosis than did euthyroid patients, as well as a higher calcium score. In patients without an altered thyroid profile, having FT4 levels in the upper quartile was associated with a higher rate of myocardial infarction in an Australian cohort study [24].

### 3.2 Clinical presentation of coronary artery disease related to hyperthyroidism

Coronary artery disease is heterogeneous and clinical presentation varies; therefore, it can be related to chronic coronary syndrome, unstable angina, acute myocardial infarction with or without ST segment elevation or sudden death [25, 26]. Even paradoxical cases without chest pain with myocardial infarction have been reported in the context of thyrotoxicosis [27]. Patients may present with cardiovascular complications such as ventricular or supraventricular arrhythmias, hemodynamic instability, cardiogenic shock and cardiorespiratory arrest [28]. The patient’s clinical picture is usually indistinguishable from a euthyroid patient with an acute coronary syndrome (ACS) of another etiology. Additionally, it usually occurs in younger patients, predominantly in women, and in some cases, the absence of classic cardiovascular risk factors is striking [25, 29, 30]. If there is a previous diagnosis of hyperthyroidism, it is necessary to evaluate adherence to antithyroid treatment and look for other extracardiovascular manifestations that suggest thyrotoxicosis [30].

There are patients with hyperthyroidism who present with chest pain, representing a diagnostic challenge [9]. In these cases, a thorough clinical history and physical examination may reveal findings that suggest thyroid disease, such as unintentional weight loss, insomnia, heat intolerance, chronic diarrhea, fine tremor, hyperreflexia, pretibial edema or exophthalmos [9]. Despite rigorous semiological analyses, some patients will not present suggestive findings, and hyperthyroidism will be detected only with an altered thyroid profile [31, 32, 33]. In general, thyrotoxicosis can occur through several mechanisms [3, 31, 34, 35]:

• Excess trophic factors that stimulate the thyroid.

• Activation and autonomous release of thyroid hormones.

• Excess passive release of thyroid hormones by autoimmune, infectious, chemical or mechanical stimuli.

• Exposure to thyroid hormones of extrathyroid origin of exogenous (pharmacological) or endogenous origin (struma ovarii or differentiated thyroid carcinoma metastasis).

Given the above, within diagnostic and therapeutic assessments, it is essential to establish the causes of thyrotoxicosis because its etiologies have different forms of treatment [36].

Within the spectrum of ACS, there is a clinical presentation with growing interest due to the uncertainty it generates, i.e., myocardial infarction with no obstructive coronary atherosclerosis (MINOCA). This represents a diagnostic challenge that leads to therapeutic uncertainty. In some cases, hyperthyroidism is identified as the underlying cause of MINOCA [10, 30, 32, 37, 38]. Bouabdallaoui *et al*. [37] reported the case of a 23-year-old patient without cardiovascular risk factors who had an acute myocardial infarction, which led to coronary catheterization that revealed a distal thrombosis of the anterior descending artery without signs of dissection or atherosclerosis. Additional studies revealed suppressed TSH and positive TSH receptor antibodies, indicating Graves’ disease. Despite not presenting other manifestations of hyperthyroidism, the patient’s cardiovascular symptoms resolved with antithyroid medical management and anti-ischemic therapy [37]. Chang *et al*. [30] presented the case of a young adult woman with previously diagnosed hyperthyroidism and poor adherence to treatment who developed chest pain with ST segment elevation. When performing coronary arteriography, a diffuse coronary spasm with extensive involvement of the anterior descending and circumflex arteries was documented. Although the condition was complicated by ventricular tachycardia and cardiorespiratory arrest, a return to spontaneous circulation was achieved with resuscitation maneuvers and intracoronary vasodilators. Cardiac function was progressively normalized once thyroid function was corrected [30]. Cases of myocardial infarction have also been reported in exogenous hyperthyroidism due to excess hormonal supplementation after thyroidectomy [34].

### 3.3 Pathophysiology of coronary artery disease related to hyperthyroidism

The pathophysiological relationship between hyperthyroidism and acute myocardial infarction can be approached from several perspectives. A higher concentration of thyroid hormones leads to increased oxygen consumption due to increased cardiac output, heart rate, myocardial contractility, and preload and decreased peripheral vascular resistance [10, 13, 39, 40, 41], which is particularly harmful in patients with previous atherosclerotic coronary artery disease [39]. Likewise, hyperthyroidism has been associated with systolic arterial hypertension, endothelial dysfunction and thrombophilia [13, 41, 42].

In patients with MINOCA, there are several theories that explain acute myocardial injury, including the imbalance between oxygen supply and demand in the context of tachyarrhythmias that condition the appearance of type 2 acute myocardial infarction. The hyperthyroid state increases the levels of catecholamines and their reactivity, generating coronary spasm of variable degrees that in turn produce acute myocardial infarction [32, 43, 44, 45]. In fact, it has been documented that patients with coronary spasm associated with hyperthyroidism tend to have a more severe presentation and worse prognosis than those who do not have hyperthyroidism [46]. This elevation of catecholamines and their receptors has also been associated with stress cardiomyopathy or Takotsubo cardiomyopathy, which in its clinical presentation is usually indistinguishable from ACS [38, 47, 48].

### 3.4 Treatment of coronary artery disease related to hyperthyroidism

Regarding the management of ACS related to hyperthyroidism, it should be the same, with anti-ischemic therapy, high-dose statin, oxygen, analgesia, invasive stratification and reperfusion therapy. The use of beta blockers is desirable as long as there are no contraindications for them. Once an altered thyroid profile is identified, its etiology should be evaluated to define the benefit of the use of antithyroid drugs such as methimazole or propylthiouracil. The opposite extreme is when the direct cause of ACS is hyperthyroidism. As mentioned above, these are usually type 2 infarcts due to tachycardiomyopathy and/or coronary spasm [45]. In this context, management should focus on ruling out atherosclerotic lesions susceptible to management, cardiac output control, antithyroid management, the identification of coronary spasms and directed management based on findings and responses [31].

## 4. Hyperthyroidism and heart failure

As has been mentioned on multiple occasions, cardiovascular function has a close relationship with thyroid status due to the direct action of T3 on cardiomyocytes, the autonomic system, vascular smooth muscle and the endothelium [16, 49, 50]. Therefore, thyroid dysfunction can be accompanied by multiple structural and/or functional alterations of the heart that ultimately lead to heart failure. Heart failure is defined as a clinical syndrome characterized by typical symptoms (such as dyspnea, ankle edema, orthopnea, and bendopnea), which may be accompanied by signs (elevated jugular venous pressure, pulmonary crackles and peripheral edema) caused by a structural cardiac abnormality or that produce a reduction in cardiac output or an increase in intracardiac pressures at rest or under stress [51]. Focusing on hyperthyroidism, the clinical presentation can vary, triggering de novo heart failure or exacerbating a previously established condition [52], presenting itself on its own or associated with other cardiovascular manifestations (tachyarrhythmias, ACS, pulmonary hypertension, and valvulopathy) [53]. Likewise, a relationship has been found with hyperthyroidism and the 2 major forms of heart failure: decreased ejection fraction (systolic) and preserved ejection fraction (diastolic). The association between heart failure and hyperthyroidism varies depending on the series evaluated, i.e., 1–7% of patients with heart failure present hyperthyroidism [54, 55, 56, 57], and approximately 10–40% of patients with hyperthyroidism present heart failure, being more frequent in those with thyrotoxicosis [58, 59].

### 4.1 Pathophysiology of heart failure related to hyperthyroidism

Taking into account that hyperthyroidism produces an increase in most cardiovascular physiological variables, it frequently presents as a particular entity called high-output heart failure. This is the result of an increase in heart rate, blood volume, stroke volume, ejection fraction and cardiac output [16, 53, 60]. In contrast, there is a decrease in peripheral vascular resistance, generating a wide pulse pressure and a decrease in mean arterial pressure. Finally, excess thyroid hormones produce an increase in the reactivity of the sympathetic system and an increase in the RAA axis and erythropoietin [61]. Therefore, there is an increase in ventricular filling pressures, increase in preload, increase in pulmonary arterial pressure, and increase in sympathetic adrenal activity, which, even in the absence of an underlying cardiovascular disease, can cause heart failure [8, 60]). Additionally, due to the high prevalence of tachyarrhythmia in hyperthyroid patients, heart failure may be secondary to tachycardiomyopathy, in which ventricular dysfunction is a consequence of persistent tachycardia and ventricular function is potentially recoverable once the arrhythmia is corrected [60]. In experimental studies with murine models of induced hyperthyroidism, elevated levels of T3 are responsible for structural cardiac changes responsible for heart failure. The hyperthyroid state produces marked hypertrophy, fibrotic cardiac remodeling due to increased sensitivity to angiotensin II, cavity dilation, ventricular wall thinning and an increased cardiomyocyte apoptosis rate, findings that may be related to the coexistence of arterial hypertension [62, 63].

### 4.2 Hyperthyroidism as a risk factor for heart failure

As previously mentioned, thyroid dysfunction behaves as a cardiovascular risk factor, even in its subclinical forms [55, 56, 61, 64]. Thus, in a meta-analysis that included more than 200 thousand patients per year, it was found that those who had subclinical hyperthyroidism had an increased risk of developing chronic heart failure, acute exacerbations and death related to heart failure [65]. Subclinical thyroid dysfunction has been related to a worse prognosis in patients with heart failure [66]; therefore, the guidelines recommend that at the time of a heart failure diagnosis, the thyroid profile of the patient should be available, and the necessary treatment should be performed as indicated, always with individualized follow-up [54].

### 4.3 Clinical presentation of heart failure related to hyperthyroidism

The clinical presentation can be varied and, in many cases, indistinguishable from heart failure without thyroid alterations [53, 60]. It is usually detected as an acute exacerbation of heart failure that can have different phenotypes based on the Stevenson classification. This is governed by clinical parameters that show the presence of congestion and/or hypoperfusion. Thus, patients may present dyspnea, orthopnea, paroxysmal nocturnal dyspnea, bendopnea, a Cheyne-Stokes respiration pattern, pulmonary rales, jugular engorgement, hepatomegaly, hepatojugular reflux, lower limb edema, ascites or signs of hypoperfusion due to alterations in consciousness, hypotension, chest pain, cyanosis, oliguria, distal coldness or mottled skin [51, 60, 67]. Diagnostic aids usually show electrocardiographic alterations such as tachyarrhythmias, hypertrophy, axis deviation and, in some cases, ischemic changes [67].

Echocardiography shows systolic and/or diastolic involvement of the left ventricle and coexistence with valvulopathies [68]. Chest X-ray usually shows varying degrees of pulmonary congestion up to frank edema and an increased cardiac silhouette [68]. Within the scenario of acute exacerbation, the measurement of serum natriuretic peptides is useful, particularly in scenarios of patients with multiple comorbidities whose main symptom is dyspnea. Elevated BNP or NT-proBNP values are consistent with the cardiac etiology of dyspnea and are also usefulness as prognostic markers [69, 70]. The use of other imaging aids, such as magnetic resonance imaging, for the diagnosis of heart failure has also been reported; however, these techniques are not generally available and have high costs, and their use is not standardized [71]. In some cases, the manifestations of hyperthyroidism, e.g., exophthalmos, goiter, xeroderma, alopecia, insomnia, heat intolerance, palpitations or diarrhea, can overcome the clinical manifestations of heart failure; therefore, an extensive history and physical examination are required to detect these manifestations [50, 72, 73].

In an observational study, Yue *et al*. [52] documented improvements in diastolic dysfunction in hyperthyroid patients with ultrasonographic methods once their endocrine alteration was corrected. This improvement was more marked in young patients under 40 years of age [52]. In a prospective study that included 30 patients with Graves’ disease, improvements in heart rate, blood pressure, arrhythmia rate and episodes of acute heart failure were documented after the initiation of antithyroid therapy. Additionally, there was a statistically significant difference in the quality of life measured and reported by the patients, being better with antithyroid therapy [74, 75, 76].

### 4.4 Treatment of heart failure related to hyperthyroidism

Although the clinical evidence regarding the benefit of therapy in subclinical hyperthyroid states is imprecise due to the variation in results found, international guidelines recommend treatment when TSH levels are lower than 0.1 mU/L and cardiovascular disease is present [3, 51, 77]. Therapy should be individualized for each patient and combined with therapy for heart failure, which depends largely on the symptoms and ejection fraction. The medical therapy for hyperthyroidism is based on 2 main pillars: the control of adrenergic activity with beta blockers and antithyroid therapy with thioamides (methimazole and propylthiouracil). Additionally, radioactive iodine or steroids can be used, particularly in thyroid storm states [61]. In some cases, the hyperthyroid state may persist despite medical therapy or be associated with thyroid neoplasia, for which surgical management is a choice [58].

In patients with heart failure and hyperthyroidism, in whom a causal relationship between the 2 is suspected, it is important to reevaluate cardiac function as thyroid function is corrected because as mentioned, structural and functional changes induced by hyperthyroidism can be reversible, making it necessary to also reevaluate the need for adjustments or withdrawal of the medication used for heart failure [67, 72, 75, 78, 79].

## 5. Hyperthyroidism and cardiac arrhythmias

Excess thyroid hormones has complex metabolic effects, particularly in the cardiovascular system and, within it, in the cardiac electrical conduction system. Subclinical hyperthyroidism is common in the general population and increases progressively with aging, being as high as 15.4% in patients over 75 years and more frequent in patients with nodular goiter, drawing attention to its association with the appearance of atrial fibrillation (AF) [80].

The most frequent clinical effects of hyperthyroidism with respect to myocardial function are sinus tachycardia and AF, which are present in up to 28% of patients [42]. In the Baladi registry [81], sinus tachycardia was found in 60.2% of patients, and AF was found in 11.7% of patients. For patients with palpitations and, especially, with de novo AF, thyroid function tests are recommended due to the previously mentioned association and prognostic value [82].

AF is the most common arrhythmia worldwide. Its prevalence increases with age; however, in patients with hyperthyroidism, in whom the prevalence of AF can reach up to 60%, it usually appears at an earlier age and in association with other types of tachyarrhythmias [83]. According to the Turan registry, comparing the presence of arrhythmias in 24-hour electrical monitoring studies, nonsustained ventricular tachycardia occurred in 18.7% of patients with Graves’ disease, and AF occurred in 30% of patients with toxic nodular goiter, all with confirmed hyperthyroidism [84].

### 5.1 Pathophysiology of hyperthyroidism related arrhythmias

Physiologically, the appearance of these tachyarrhythmias occurs because in hyperthyroidism, excess thyroid hormones alter the function of cardiac B1 adrenergic and muscarinic receptors, resulting in an increase in sympathetic function, producing tachycardia and a decrease in the refractory period. Additionally, it has been documented in mice that thyroid hormones increase the expression of atrial ion channel messenger RNA, increasing potassium receptors 1.5 times, which increases intracellular potassium intake, ultimately leading to a decrease in the atrial refractory period [85]. Another mechanism involved is the increase in adrenergic stimulation associated with the hyperthyroid state, which favors the appearance of atrial premature beats that trigger acute episodes of AF in a dilated and remodeled atrial substrate associated with volume overload and stimulation by the RAA axis [86]. These alterations clinically manifest as palpitations, evident in the 24-hour electrocardiographic record, i.e., a constant increase in heart rate during the day associated with an exaggerated response during exercise [87].

### 5.2 Electrocardiogram and hyperthyroidism

On electrocardiography, the most common abnormalities are sinus tachycardia and shortening of the PR interval associated with an increase in the duration of the P wave due to a prolongation in intra-atrial conduction [87]. Intraventricular conduction is altered in up to 15% of patients due to the presence of a right bundle branch block. The prevalence of AF and other less common supraventricular arrhythmias ranges from 2 to 60%, approximately 5 times more frequent than that in the euthyroid population [88].

### 5.3 Treatment of hyperthyroidism related arrhythmias

The mainstay of treatment for tachyarrhythmias induced by hyperthyroidism is a beta-blocker and antithyroid agent (propylthiouracil or methimazole), with propranolol being the first choice because it blocks the peripheral conversion of T4 to T3 [8]. In patients where beta-blocker therapy is contraindicated, other management options include calcium channel blockers such as diltiazem or verapamil. However, these agents should be avoided in those with a reduced ejection fraction or hemodynamic instability because of a strong negative inotropic effect [89].

Amiodarone can be used in the acute setting in selected cases in which a rhythm control strategy is desired, taking special care with its use given the possibility of inducing additional alterations in thyroid function; therefore, its use always needs to be concomitant with antithyroids [90, 91]. However, unless there is hemodynamic instability, rhythm control is not a priority measure because early cardioversion, when compared with a conservative strategy, is not superior in achieving a greater proportion of sinus rhythm at 14 days, particularly in this population. Approximately two-thirds of patients revert to a sinus rhythm 8 to 10 weeks after returning to the euthyroid state [91, 92].

In those patients who persist with AF despite being euthyroid, rate control is initially preferred, with rhythm control with class IA, IC, and III antiarrhythmics as a second option. Other measures used when adequate rhythm control is not achieved include electrical cardioversion in those patients who persist with AF after 8 to 10 weeks of returning to the euthyroid state [90].

Amiodarone, a benzofuranic antiarrhythmic drug rich in iodine, can induce thyrotoxicosis in 7–15% of patients; this is an important problem due to its potential negative impact on cardiac function in patients with underlying tachyarrhythmia. Amiodarone-induced thyrotoxicosis (AIT) is a condition induced by iodine in patients with abnormal thyroid (type 1) or resulting from acute thyroiditis in a “healthy” thyroid (type 2). Determining the type of AIT is a diagnostic dilemma because the characteristics of both types may be present in some patients. When suspected, treatment with amiodarone should be suspended; however, recently, some studies have shown that amiodarone can be continued or reintroduced in patients with a history of type 2 AIT [91]. In patients who require management with amiodarone and have no underlying thyroid disease, thyroid function tests should be conducted prior to the start of such therapy, at 3 months after starting and then every 3 to 6 months; in those with thyroid disease, these tests should be performed prior to the start of therapy, at 1 month after starting and then every 3–6 months based on disease evolution [93].

AF in patients with hyperthyroidism also increases the risk of developing thromboembolic cerebrovascular events. In this regard, current guidelines establish the use of CHA2DS2-VASC to define the benefits of anticoagulation; however, in the case of hyperthyroidism, elevated levels of thyroxine theoretically increase the risk of a thrombotic event due to metabolic alterations associated with increased levels of fibrinogen and coagulation factors VII and IX [87, 91]; therefore, having AF associated with hyperthyroidism implies an additional increase in the risk of thromboembolic events. However, a study by Chan *et al*. [94] with 9727 patients with nonvalvular AF reported that hyperthyroidism does not independently increase the risk of thrombotic events. Thus, the current recommendations issued by the American College of Chest Physicians are that in these patients, anticoagulation decisions should be based on the CHA2DS2-VASC score [83, 95]. In the younger population, in whom the CHA2DS2-VASC score may have a numerically lower result, the predictive capacity of embolic events seems to be lower than that in the older population. Therefore, some groups have recommended other strategies, such as using transesophageal echocardiography to search for thrombotic environments, a strategy that currently requires validation and additional evaluation [96].

In the past, warfarin was the recommended anticoagulant, and in some scenarios, ASA was used as antiplatelet therapy. However, currently, the ability to reduce embolic events with ASA is not optimal compared to oral anticoagulants, maintaining a bleeding profile similar to these [97]. In the case of direct oral anticoagulants, there are recent observational studies that suggest an acceptable safety profile as an alternative to the use of warfarin [98]. Importantly, subjects who receive electrical cardioversion and in whom the duration of symptoms is greater than 12 hours should receive oral anticoagulation independent of the CHA2DS2-VASC score for at least 4 weeks to mitigate the risk of embolic events [99].

## 6. Hyperthyroidism and perioperative cardiovascular risk

Currently and based on our review, no clinical trials have evaluated cardiovascular or perioperative outcomes in hyperthyroid patients undergoing surgery; therefore, it is necessary to extrapolate the aforementioned hemodynamic changes of the disease and add them to those pertaining to anesthesia and surgery to establish preoperative cardiovascular risk.

The probability of adverse outcomes for an uncontrolled hyperthyroid patient who is undergoing surgery is higher than that for those who are controlled and receive treatment prior to surgery. Perioperative complications derived from hyperthyroidism are highly variable given the systemic effect of thyroid hormones. Mainly, those of greatest concern are cardiovascular complications secondary to the hyperdynamic circulatory state. Vasodilation, decreased systemic vascular resistance and increased cardiac output lead to the exacerbation or precipitation of myocardial ischemia or high-output heart failure [100, 101]. The incidence of AF is 10% to 20% in patients with overt hyperthyroidism and subclinical hyperthyroidism [101, 102].

Other situations related to the hypercatabolic state of severe hyperthyroidism are anorexia, malnutrition, hypoalbuminemia, hyperthermia, electrolyte disorders (hyponatremia, hypercalcemia) and myopathy manifesting as generalized muscle and respiratory weakness that increase surgical risk and postoperative complications [101].

The most feared perioperative risk is a thyroid storm, an infrequent but potentially fatal manifestation, with an incidence rate ranging from 8–25% [103, 104]. Basically, it is characterized by the dysfunction of 1 or several organs associated with thyrotoxicosis. Clinically, it manifests as hyperthermia, tachycardia and an altered state of consciousness, which can lead to cardiovascular collapse and death. These usually occur in the intraoperative or postoperative period [101]. Most of the articles published regarding perioperative management include a systematic evaluation of thyroid function in patients with a history of hyperthyroidism or compatible symptoms and the optimization of their cardiovascular status whenever possible [102, 103, 104, 105].

In subclinical hyperthyroidism and in very specific cases, surgery can be performed. However, for elective surgeries and cases with significant surgical risks, surgery should be delayed until the euthyroid state is reached, which can be achieved after a few weeks of treatment and with adequate follow-up. With the objective of controlling chronotropism and cardiovascular function, beta-blockers are the main treatment, titrated based on the response [101].

In cases of urgent or emergent surgeries, invasive cardiovascular monitoring is required, as well as premedication with beta-blockers and antithyroid drugs and the possibility of glucocorticoids and/or exchange resins. The preferred beta-blockers are propranolol, which blocks the conversion of T4 to T3 through the selective inhibition of D1 [3, 106], or esmolol, which can be administered intravenously and rapidly titrated because it has a short life. Furthermore, studies with metoprolol have reported adequate outcomes [103, 107].

Additionally, antithyroid drugs, which decrease hormone synthesis, can be administered orally or intrarectally. In cases requiring rapid stabilization of thyrotoxicosis, solutions with inorganic iodine (lugol or potassium iodide) can be used 1 hour after the administration of the antithyroid drug; they are commonly administered in 3–5 drops, 3 or 4 times per day [3, 102]. Hydrocortisone at a dose of 100 mg every 8 hours IV for 3 days, dexamethasone 2 mg orally or IV every 6 hours, and betamethasone 0.5 mg IM or IV every 6 hours are other therapeutic options [101, 102]. Finally, cholestyramine is an additional modality that can be used to rapidly reduce thyroid hormone levels in thyrotoxic patients. At a dose of 4 g 4 times per day, cholestyramine decreases the levels of circulating hormones by binding to thyroid hormones in the intestine, decreasing their reabsorption and enterohepatic recirculation [3, 102]. For patients who are intolerant to beta blockers, calcium antagonists are an option, as explained above [102].

## 7. Echocardiographic findings in hyperthyroidism

There are multiple echocardiographic findings in patients with thyroid disease, and these findings are subject to the aforementioned pathophysiological changes. These alterations can be detected by echocardiography even in the early stages of the disease and, for practical purposes, can be subdivided into those that occur during the clinical or subclinical stage [108].

### 7.1 Subclinical stage

In the initial stages of the disease, both structural and functional changes are observed. The most notable structural changes are due to alterations in wall thickness, especially due to an increase in both the relative wall thickness and the left ventricular mass [108].

Within the changes in systolic function, patients generally have an ejection fraction within the normal range or slightly increased; therefore, the most important parameter to detect functional changes during the subclinical stage is a decrease in longitudinal strain [108].

Diastolic function can generally be altered by a type I relaxation disorder [109] in which there is a loss of left ventricle elastic recoil in early diastole, a reduction in the suction force of the LV and a low pressure gradient between the opening of the mitral valve and ventricle, leading to a delay in the opening of the mitral valve (E wave <0.8 m/sec) and an increased isovolumic relaxation time with a prolonged deceleration time [110].

### 7.2 Clinical stages

During this stage, the structural changes can vary from the presence of concentric hypertrophy of the left ventricle to left ventricular growth with the subsequent development of dilated cardiomyopathy, which is usually reversible once the patient returns to the euthyroid state; however, one-third of these patients usually persist with these alterations [111, 112]. For reasons that are not very clear, mitral valve prolapse has been found in 16.5 to 25% of patients with Graves’ disease, of whom 71% have mitral insufficiency and 63% have tricuspid insufficiency [52, 112].

In relation to systolic function, an increase in the ejection fraction of the left ventricle is observed due to its hyperdynamic state [113], and as the disease progresses, a greater deterioration of diastolic function is observed due to a progressive loss of the distensibility of the left ventricle with an increase in the filling pressures, causing a marked increase in the early inflow velocity (E wave >1.5 m/sec) with a decrease in the atrial active stage of the cardiac cycle (E/A >2 m/sec) [113]. Additionally, there is a shortening of the isovolumic relaxation time, and the filling pressure of the left atrium increase, which determines the appearance of left atrial dilation [52].

Among other findings, it has been documented that up to 65% of patients with Graves’ disease can present pulmonary hypertension [114]. Therefore, thyroid disease should be considered in the differential diagnosis of primary pulmonary hypertension with a progressive component that leads to right heart failure and premature death [114].

## 8. Hyperthyroidism and vascular complications

### 8.1 Systemic vasculature and hyperthyroidism

Systemic arterial hypertension is a major global health concern affecting around 25% of population [17]. Many of the previously mentioned complications related to hyperthyroidism, particularly myocardial infarction and heart failure, are partially due to effects of thyroids hormones in vasculature. It is well known that hyperthyroidism is a cause of secondary hypertension (predominantly systolic) by activating RAA axis and accelerating atherosclerosis [115, 116]. This results in augmentation of metabolic rate, cardiac preload and ventricular contractility. Nevertheless, T3 induces local vasodilatation seeking thermogenesis resulting in decreased systemic vascular resistance and diastolic pressure.

Arterial stiffness (decreased elastic responsiveness of vessel wall to ventricular pressure wave), determined by elastin-to-collagen ratio, is a parameter promoted by most of cardiovascular risk factors and works as a surrogate marker of atherosclerosis [117]. Clinical and preclinical evidence supporting the role of either overt or subclinical hyperthyroidism on arterial stiffness is scarce and in some aspects contradictory, as reviewed by Anagnostis *et al*. [118], but some small observation studies points towards a direct relation between these two variables [119]. Also, it seems that non-selective betablockers and restoring thyroid function improves arterial stiffness, systolic blood pressure and adverse cardiovascular events [115]. Some of the limitations of these studies are that most of patients included had Grave’s disease, which is a autoimmune phenomenon that could affect the vasculature through other mechanisms [118, 120].

### 8.2 Pulmonary vasculature and hyperthyroidism

The two main complications of hyperthyroidism in pulmonary circulation are pulmonary hypertension and pulmonary embolism, as it will be discussed.

The prevalence of pulmonary hypertension in hyperthyroidism is relatively high, with some studies varying between 36 and 65% [115, 121], most of them mild or asymptomatic. As in other causes of pulmonary hypertension, this complications is a marker of poor prognosis, mainly because of right heart failure [9]. Some proposed mechanisms that explain pulmonary hypertension in hyperthyroidism are left side failure, hyperdynamic circulation and/or remodeling effects of pulmonary vasculature. Treating and correcting thyroid profile with anti-thyroid drugs seems to decrease pulmonary artery systolic pressure [121].

Several observational studies have stated the relationship between hyperthyroidism and pulmonary embolism [122, 123, 124, 125, 126], increasing the risk of this complication in a 2 fold way. Even more, other thrombotic complications such as deep vein thrombosis and cerebral venous thrombosis have be associated with hyperthyroidism. Increased thyroid function impacts on Virchow’s triad by shortening activated partial thromboplastin time, higher fibrinogen and factor VIII levels and lower plasma fibrinolytic capacity resulting in hypercoagulable state [127, 128]. Hemodynamic changes largely discussed result in blood turbulence and endothelial damage [127, 129]. Although, some trials have failed to show a higher risk of pulmonary embolism in overt or subclinical hyperthyroidism, particularly in the elderly or hospitalized patients [130, 131]. Given this scenario, most experts recommend testing thyroid function in unprovoked pulmonary embolism and considering normalizing its function as part of the prevention of thrombotic events recurrence [123, 124, 132].

## 9. Conclusions

Subclinical and overt hyperthyroidism increases the risk of different cardiac conditions, which lead to an increase in cardiovascular morbidity and mortality in this population. The recognition and detection of these manifestations in patients with thyroid disease, as well as the reinforcement of the need for thyroid function tests in patients with cardiovascular disease, are very important.

## Abbreviations

ACS, acute coronary syndrome; AF, atrial fibrillation; ANP, atrial natriuretic peptide; D1, Type 1 deiodinase; D2, Type 2 deiodinase; D3, type 3 deiodinase; MINOCA, myocardial infarction with no obstructive coronary atherosclerosis; RAA, the renin-angiotensin-aldosterone; T2, diiodothyronine; T3, triiodothyronine; T3r, reverse triiodothyronine; T4, thyroxine; TRH, thyrotropin-releasing hormone; TSH, thyroid-stimulating hormone.
